# Supplementary material for: Mechanistic and microkinetic study of non-oxidative methane coupling on a single-atom iron catalyst
Source: Commun Chem. 2020 May 8;3:58. doi: 10.1038/s42004-020-0306-1 (PMC9814405; doi:10.1038/s42004-020-0306-1)
Supplement: Supplementary file 1 — Supplementary Information [file 42004_2020_306_MOESM1_ESM.pdf]

Supplementary Information for

Mechanistic and microkinetic study of non-oxidative methane coupling on a single-atom iron catalyst

Seok Ki Kim,[1,2,*] Hyun Woo Kim,[3] Seung Ju Han,[1] Sung Woo Lee,[1] Jungho Shin,[3] Yong Tae Kim[1,*]

[1] C1 Gas & Carbon Convergent Research Center, Korea Research Institute of Chemical Technology, Daejeon 34114, Republic of Korea
[2] Advanced Materials and Chemical Engineering, University of Science and Technology, Daejeon 341113, Republic of Korea
[3] Chemical Data-Driven Research Center, Korea Research Institute of Chemical Technology, Daejeon 34114, Republic of Korea

*Corresponding authors: skkim726@krict.re.kr, ytkim@krict.re.kr

Contents

Supplementary Figure 1. Transition state scaling and BEP scaling. 2

Supplementary Figure 2. Change in free energies of activation 3

Supplementary Figure 3. Degree of rate control for all states 4

Supplementary Table 1. Hydrocarbon distributions obtained using the Fe@CRS catalyst under various reaction conditions. 5

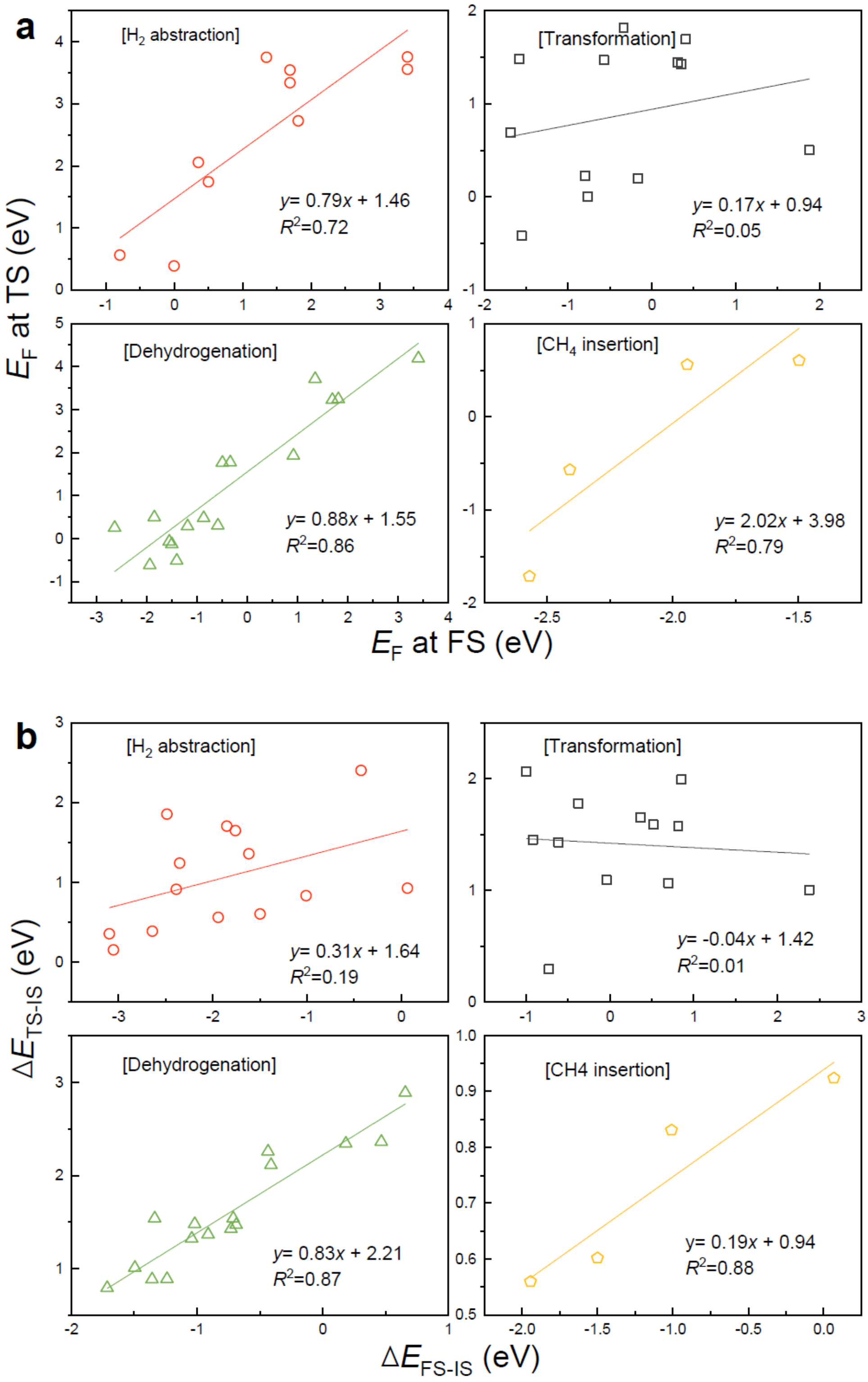

Supplementary Figure 1. a, Transition-state scaling with formation energy at final state (E_F at FS). **b,** Brønsted-Evans-Polanyi (BEP) relation.

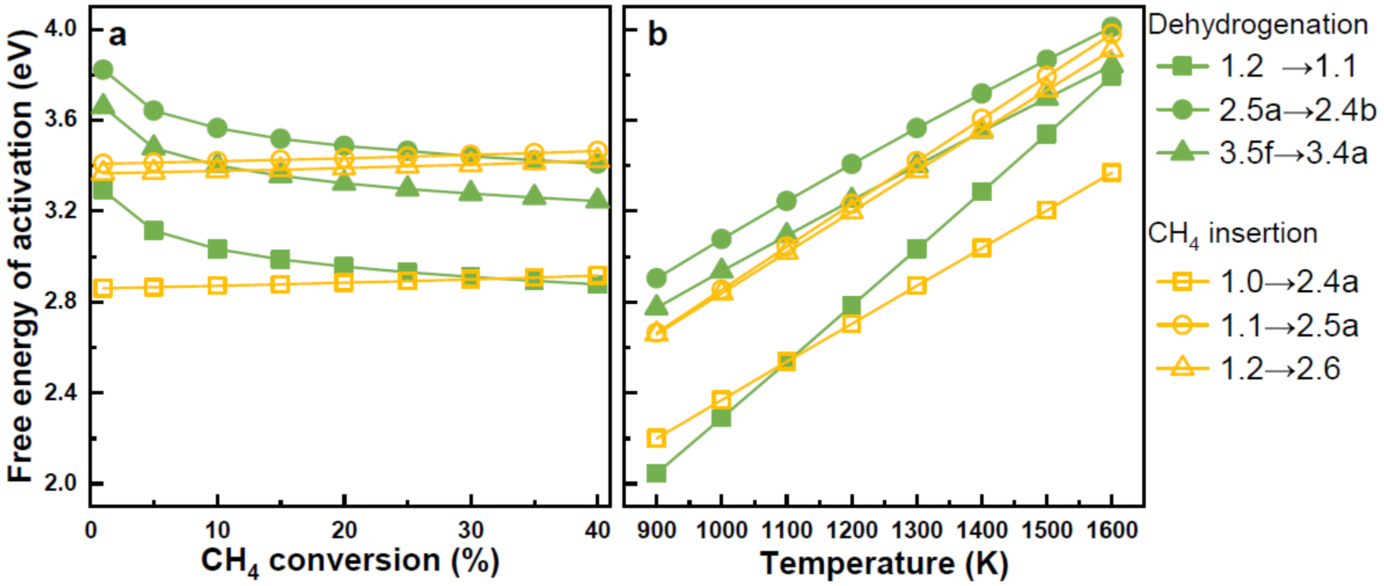

Supplementary Figure 2. Change in free energies of activation for dehydrogenation and CH_4 insertion reactions. a, Effect of CH_4 conversion at 1300 K and 1 bar. **b,** Effect of reaction temperature at CH_4 conversion of 10% and 1 bar. CH_4 was assumed to be converted to CH_3 and 1/2 H_2.

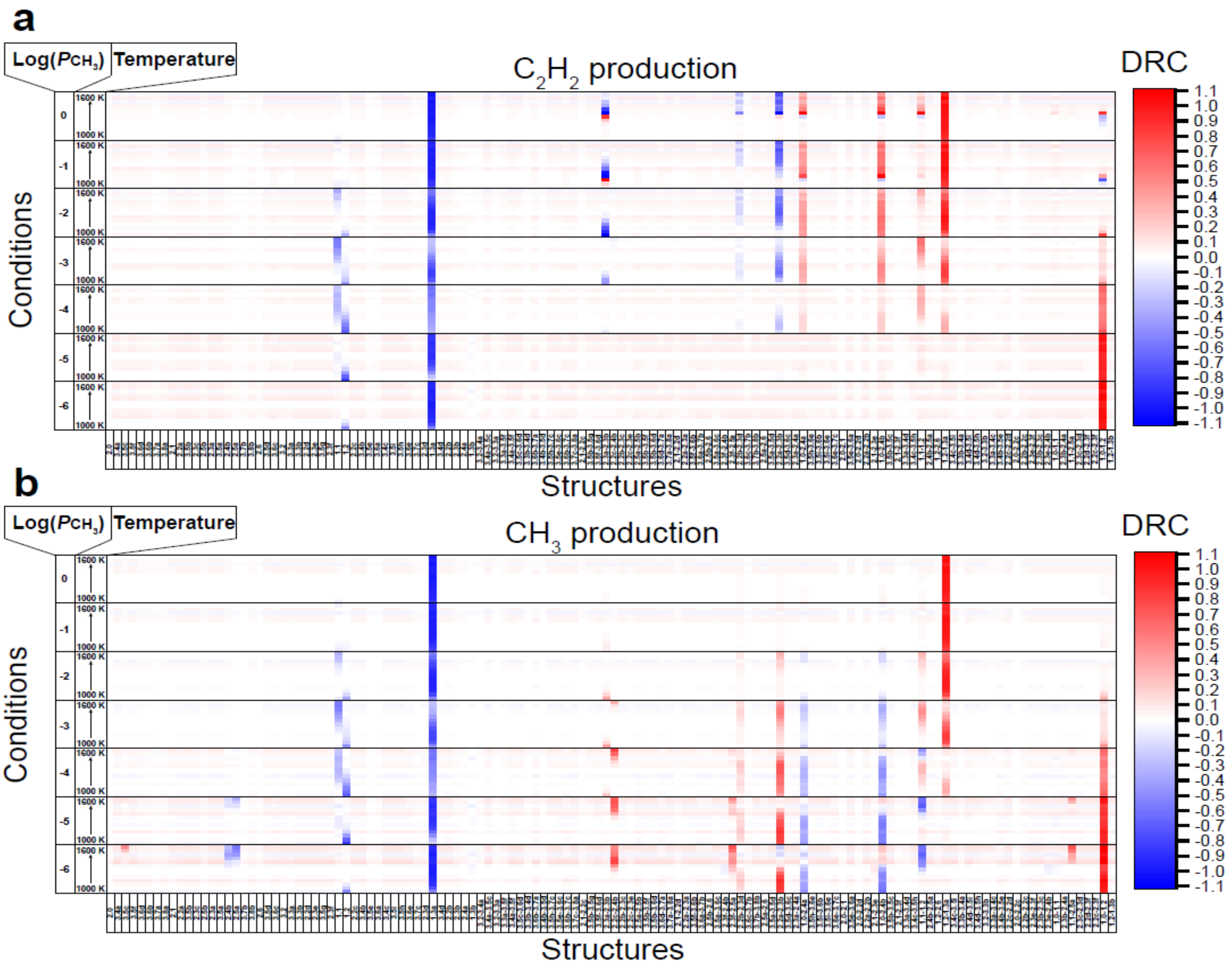

Supplementary Figure 3. Degree of rate control calculated for all states calculated under different reaction conditions. **a,** DRC values for the production of C_2H_2. **b,** DRC values for the production of CH_3.

Supplementary Table 1. Hydrocarbon distributions obtained using the Fe@CRS catalyst under various reaction conditions.

t_{gas}[a] (s)	t_{cat}[b] (s)	X[c] (%)	Selectivity (%)										
			C_2H_6	C_2H_4	C_2H_2	C_3	C_4	C_5	C_6H_6	C_7H_8	$C_{10}H_8$	Alkyl benzenes	Coke
1.7	0.0	1.0	20.0	38.4	8.9	8.0	9.5	1.8	2.0	0.0	0.0	3.1	8.3
2.3	0.0	1.8	13.1	34.5	12.0	7.9	12.8	2.8	6.0	1.4	0.9	3.2	5.4
2.8	0.0	2.9	9.6	32.3	14.8	7.6	12.7	2.8	11.6	2.9	3.2	2.4	0.0
11.3	0.0	24.3	1.0	15.9	11.3	1.2	1.1	0.5	22.4	1.7	11.4	3.3	30.3
1.8	0.0	4.7	6.0	28.1	16.9	6.1	9.5	2.1	9.9	2.3	2.2	3.8	13.0
2.4	0.1	7.3	3.9	23.6	17.3	4.5	1.8	1.8	12.5	2.7	3.7	3.8	24.5
11.7	0.3	26.6	0.8	14.9	11.4	1.0	1.0	0.5	19.0	1.3	5.9	2.5	41.8
40.4	1.1	42.3	0.8	9.1	4.6	0.3	0.2	0.1	13.1	0.4	5.5	1.0	64.9
1.8	0.1	6.5	4.4	26.9	18.7	5.0	7.6	1.6	11.8	2.5	3.3	2.8	15.3
2.4	0.1	9.5	2.8	22.1	18.1	3.6	5.2	1.4	12.8	2.4	4.3	3.1	24.2
3.0	0.2	11.0	2.3	21.3	18.5	3.3	4.5	1.2	15.3	2.6	5.5	3.9	21.5
12.0	0.6	27.2	0.8	14.2	11.0	0.9	0.8	0.3	16.5	1.1	4.7	2.0	47.6
41.5	2.1	41.5	0.8	9.5	4.7	0.3	0.2	0.1	12.5	0.4	4.9	1.0	65.6
2.2	0.2	7.9	3.4	24.6	18.0	4.4	6.2	0.0	10.1	2.1	2.5	5.3	23.4
2.9	0.3	10.6	2.2	20.4	17.1	3.2	4.5	0.0	10.4	1.8	3.0	4.5	32.9
3.7	0.3	12.2	2.0	19.3	17.4	2.8	3.8	0.0	11.9	1.9	4.0	3.8	33.2
14.6	1.2	26.3	0.8	13.8	11.3	1.0	0.9	0.0	13.4	0.9	4.5	2.1	51.5
50.5	4.3	40.4	0.8	10.0	5.1	0.3	0.2	0.0	11.2	0.4	4.1	1.1	66.8

[a] Gas-phase residence time
[b] Catalyst contact time
[c] Methane conversion
