# Supplementary material for: Mechanistic and microkinetic study of non-oxidative methane coupling on a single-atom iron catalyst
Source: Commun Chem. 2020 May 8;3:58. doi: 10.1038/s42004-020-0306-1 (PMC9814405; doi:10.1038/s42004-020-0306-1)
Supplement: Supplementary file 2 — Description of Additional Supplementary Files [file 42004_2020_306_MOESM2_ESM.pdf]

Description of Additional Supplementary Files

File Name: Supplementary Data 1
Description: Structures, formation energies, and vibrational frequencies of species used in microkinetic analysis for the non-oxidative methane coupling reaction. Formation energies were calculated using electronic energies of CH4, H2, and structure '1.0' as references. Color reference: brown, C; white, H; silver, Si; red, O; gold; Fe.

File Name: Supplementary Data 2
Description: Elementary reactions involved in the microkinetic modelling. * and _g denote surface and gas-phase species, respectively.
