# Supplementary material for: Mechanistic and microkinetic study of non-oxidative methane coupling on a single-atom iron catalyst
Source: Commun Chem. 2020 May 8;3:58. doi: 10.1038/s42004-020-0306-1 (PMC9814405; doi:10.1038/s42004-020-0306-1)
Supplement: Supplementary file 3 — Supplementary Data 1 [file 42004_2020_306_MOESM3_ESM.pdf]

Supplementary data 1. Structures, formation energies, and vibrational frequencies of species used in microkinetic analysis for the non-oxidative methane coupling reaction. Formation energies were calculated using electronic energies of CH_4, H_2, and structure '1.0' as references. Color reference: brown, C; white, H; silver, Si; red, O; gold; Fe.

Notation	Structure	Formation energy (eV)	Vibrational frequencies (cm^{-1})
CH_4	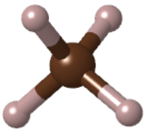	0.000	3450, 3333, 2963, 2801, 2151, 1703, 1272, 502, 242, 73
H_2	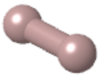	0.000	4422, 92, 45
C_2H_2	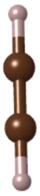	4.079	3415, 3319, 1994, 767, 765, 618, 613, 108, 89
C_2H_4	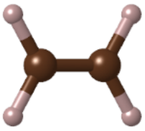	2.139	3152, 3122, 3070, 3059, 1640, 1458, 1358, 1224, 1041, 948, 935, 823, 10, 4
C_2H_6	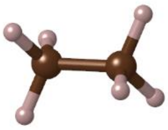	0.729	3018, 3003, 2947, 2945, 2754, 2731, 1492, 1488, 1396, 1389, 1302, 1230, 957, 820, 819, 326, 257, 118, 114
CH_3	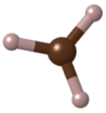	2.263	3215, 3196, 3036, 1395, 1392, 524, 172, 109, 60
1.0	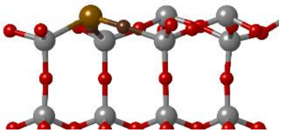	0.000	

Notation	Structure	Formation energy (eV)	Vibrational frequencies (cm^{-1})
1.1	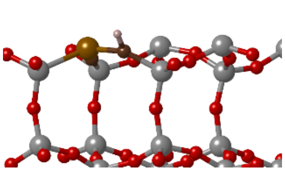	-1.402	2675, 851, 406
1.2	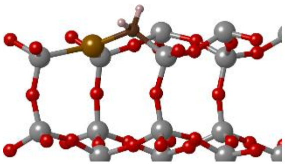	-2.639	2886, 2707, 1287, 847, 807, 398
1.3a	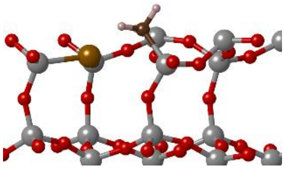	-2.723	3002, 2964, 2820, 1462, 1428, 1268, 776, 714, 551, 248, 213, 149
1.3b	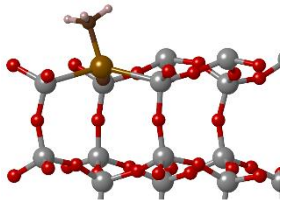	-1.982	3049, 3013, 2920, 1424, 1396, 1125, 627, 584, 429, 191, 95, 69
2.0	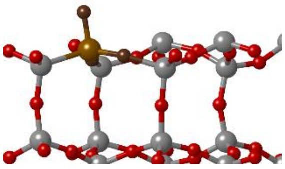	3.404	844, 140, 125
2.1	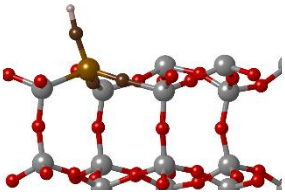	1.690	3065, 845, 736, 668, 143, 66
2.2a	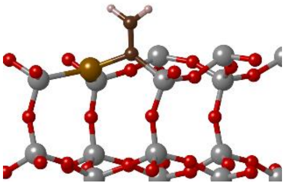	-0.865	3016, 2964, 1494, 1001, 966, 934, 540, 175, 73

Notation	Structure	Formation energy (eV)	Vibrational frequencies (cm^{-1})
2.2b	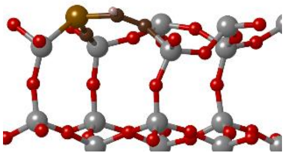	-0.165	3014, 2828, 1474, 1236, 1071, 837, 676, 572, 503, 272, 232, 200
2.2c	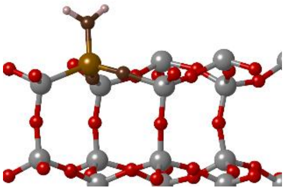	0.353	3096, 2968, 1337, 848, 655, 570, 126, 66
2.2d	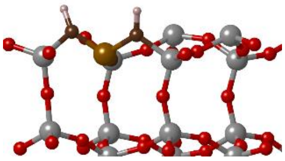	0.310	2904, 2883, 899, 852, 612, 508
2.3a	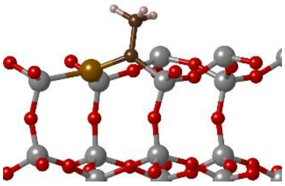	-0.583	2949, 2920, 2886, 1445, 1439, 1351, 989, 863, 655, 168, 142, 69
2.3b	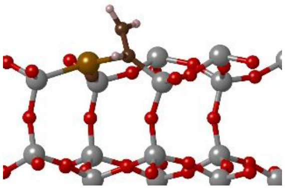	-1.578	3114, 3000, 2860, 1476, 1317, 1197, 1016, 916, 847, 660, 577, 384, 341, 283, 105
2.3c	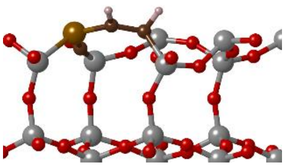	-0.764	2988, 2880, 2848, 1424, 1262, 1156, 1105, 1009, 760, 640, 560, 474, 308, 225, 165
2.3d	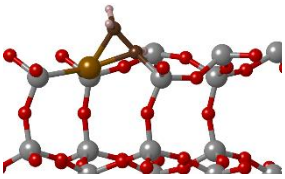	-1.684	3095, 2971, 2913, 1460, 1221, 987, 912, 865, 800, 632, 353, 192

Notation	Structure	Formation energy (eV)	Vibrational frequencies (cm^{-1})
2.3e	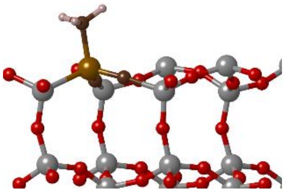	-0.065	3071, 3007, 2929, 1421, 1399, 1127, 639, 613, 451, 156, 99, 78
2.3f	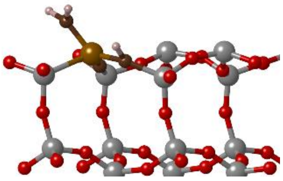	-0.792	3098, 2951, 2723, 1343, 951, 869, 676, 626, 571, 413, 188, 135
2.4a	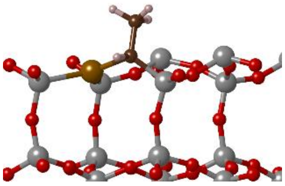	-1.941	3013, 2947, 2916, 2655, 1475, 1464, 1389, 1202, 1057, 986, 918, 873, 634, 409, 307, 220, 145, 62
2.4b	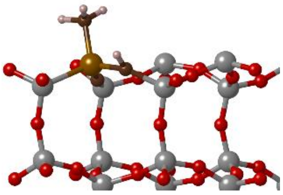	-1.497	3062, 3020, 2935, 2727, 1434, 1403, 1144, 917, 691, 672, 498, 444, 185, 122, 86
2.5a	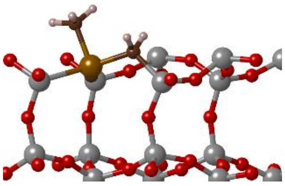	-2.409	3087, 3019, 2908, 1451, 1407, 1187, 769, 721, 449, 260, 179, 143
2.5b	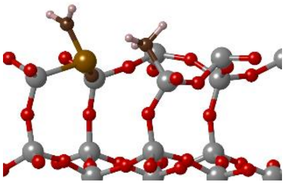	-1.553	3094, 2936, 1322, 806, 669, 527, 457, 167, 117
2.6	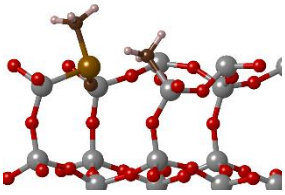	-2.570	2986, 2950, 2883, 1462, 1435, 1240, 799, 704, 564, 302, 184, 152

Notation	Structure	Formation energy (eV)	Vibrational frequencies (cm^{-1})
3.2	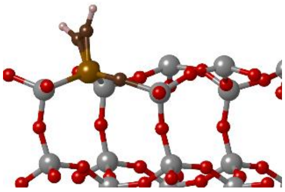	1.348	3114, 2959, 1265, 1088, 1004, 845, 801, 686, 611, 295, 235, 107
3.3a	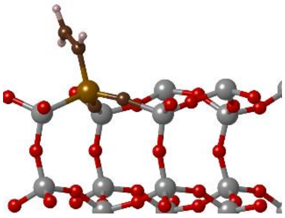	1.814	3111, 3026, 2985, 1518, 1363, 1203, 943, 931, 900, 464, 392, 240, 108, 84, 56
3.3b	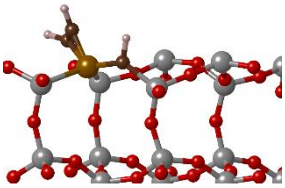	0.175	3076, 2967, 2861, 1286, 1132, 1020, 916, 850, 810, 758, 645, 529, 316, 293, 132
3.4a	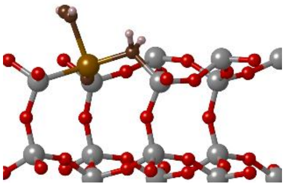	0.923	3378, 3282, 1899, 758, 703, 650, 553, 129, 98, 88, 49, 31
3.4b	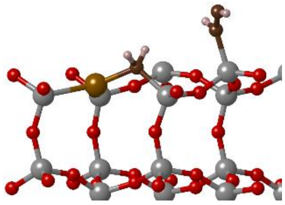	-0.199	3139, 2975, 1584, 1149, 864, 848, 570, 547, 272, 166, 85
3.4c	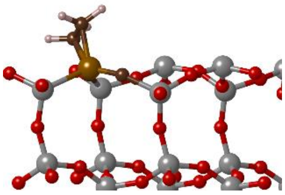	0.148	3118, 3046, 3025, 2973, 1438, 1424, 1205, 1118, 1006, 959, 900, 810, 673, 557, 401, 244, 156, 107
3.4d	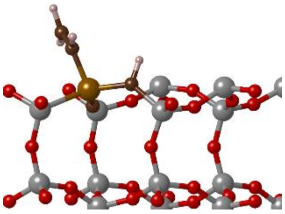	1.084	3119, 3026, 2937, 2866, 1517, 1361, 1206, 949, 935, 906, 895, 508, 416, 395, 246, 155, 94, 36

Notation	Structure	Formation energy (eV)	Vibrational frequencies (cm^{-1})
3.5a	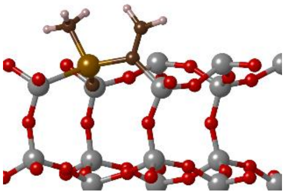	-0.497	3090, 3033, 2938, 1427, 1405, 1126, 657, 617, 442, 250, 149, 115
3.5b	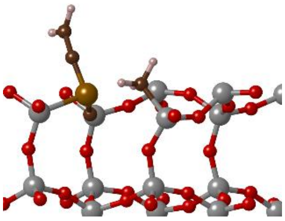	0.039	3132, 3055, 1487, 1319, 876, 724, 521, 356, 315, 171, 94, 70
3.5c	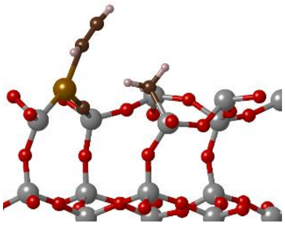	-0.335	3358, 2873, 1787, 754, 678, 528, 486, 216, 192, 125, 47, 35
3.5d	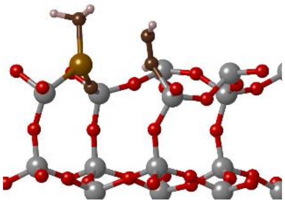	1.880	3130, 2961, 1561, 1152, 865, 855, 571, 551, 289, 194, 121, 32
3.5e	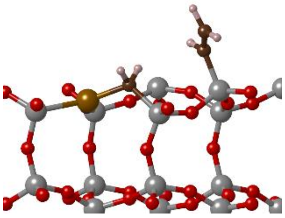	-2.378	3118, 3044, 3023, 1591, 1419, 1277, 1021, 1005, 959, 574, 509, 304, 163, 111, 77
3.5f	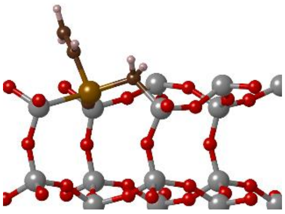	-0.571	3111, 3033, 2895, 1565, 1386, 1187, 920, 846, 816, 457, 432, 312, 226, 124, 68
3.5g	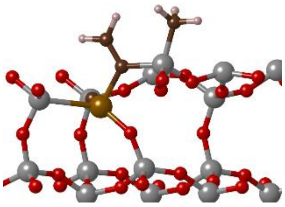	0.406	3087, 3024, 1480, 994, 973, 920, 584, 302, 114

Notation	Structure	Formation energy (eV)	Vibrational frequencies (cm^{-1})
3.5h	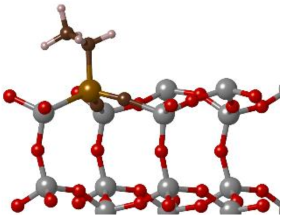	0.503	3034, 3010, 2974, 2919, 2901, 1473, 1464, 1422, 1383, 1209, 1128, 960, 926, 880, 606, 421, 238, 189, 96, 84, 71
3.5i	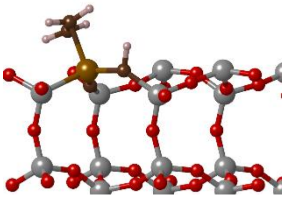	-0.164	3173, 3142, 3082, 3060, 2792, 1524, 1448, 1244, 1217, 971, 966, 910, 878, 820, 562, 435, 341, 191, 122, 90, 75
3.6a	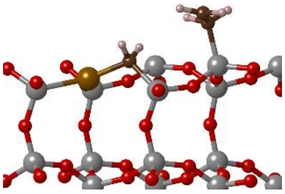	-1.937	3160, 3065, 2979, 2900, 1445, 1420, 1223, 1124, 1016, 928, 700, 568, 486, 255, 220, 176, 90, 72
3.6b	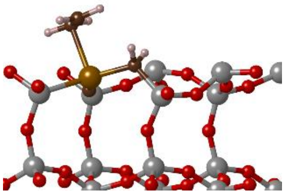	-1.184	3172, 3143, 3072, 3064, 1587, 1454, 1328, 1223, 1031, 990, 958, 823, 377, 196, 114, 95, 37
3.6c	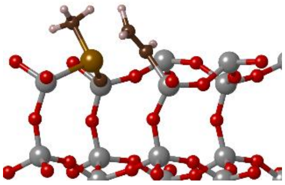	-0.930	3125, 3046, 3037, 1525, 1374, 1263, 1023, 975, 915, 583, 544, 345, 304, 208, 98
3.6d	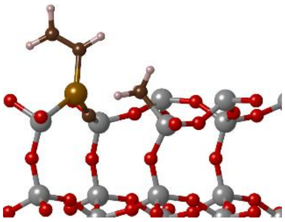	-0.744	3084, 3015, 2995, 1535, 1374, 1221, 972, 941, 929, 499, 388, 193, 104, 92, 49
3.6e	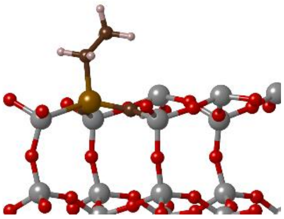	-0.728	3019, 2988, 2979, 2925, 2880, 2874, 1470, 1463, 1420, 1381, 1217, 1140, 1003, 995, 929, 885, 661, 568, 407, 244, 224, 156, 49, 25

Notation	Structure	Formation energy (eV)	Vibrational frequencies (cm^{-1})
3.7a	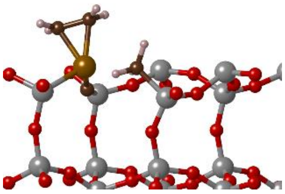	-1.870	3127, 3083, 3048, 3005, 1498, 1441, 1206, 1194, 937, 897, 859, 792, 618, 428, 340, 147, 119, 91
3.7b	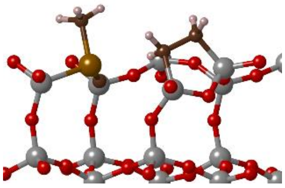	-2.978	2995, 2978, 2956, 2940, 1446, 1434, 1238, 1225, 1072, 1028, 882, 783, 721, 582, 554, 308, 201, 166
3.7c	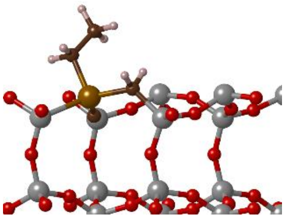	-1.847	3021, 3004, 2980, 2922, 2890, 2870, 2769, 1476, 1471, 1410, 1383, 1308, 1213, 1130, 956, 919, 907, 886, 865, 596, 409, 395, 241, 202, 122, 89, 56
3.8a	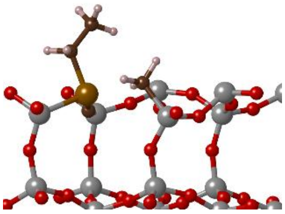	-1.663	3040, 2999, 2977, 2946, 2772, 1481, 1464, 1414, 1383, 1172, 1111, 981, 945, 923, 545, 319, 206, 119, 104, 57
3.8b	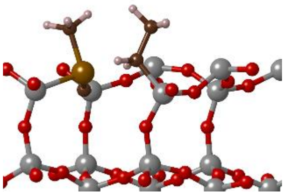	-1.771	3033, 3010, 2957, 2820, 2714, 1488, 1486, 1471, 1399, 1205, 1154, 976, 954, 908, 855, 559, 313, 272, 168, 154, 77
1.0-1.2	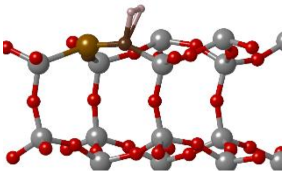	0.387	3256, 2578, 1205, 696, 503, 336
1.0-2.4a	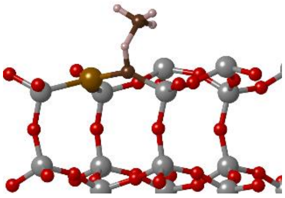	0.559	3158, 3112, 2999, 2003, 1437, 1414, 1140, 989, 784, 557, 311, 142, 94, 60

Notation	Structure	Formation energy (eV)	Vibrational frequencies (cm^{-1})
1.0-2.4b	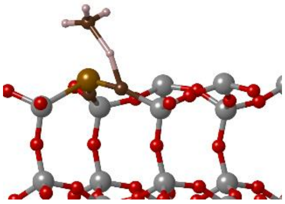	0.601	3070, 3026, 2938, 1673, 1440, 1431, 1136, 856, 692, 542, 177, 137, 106, 93
1.1-1.2	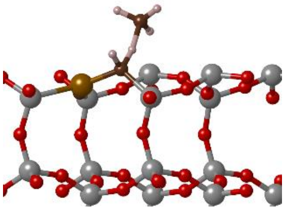	-0.516	3153, 3143, 3004, 1404, 1400, 1251, 1089, 1013, 526, 475, 397, 101, 69, 34
1.1-2.5a	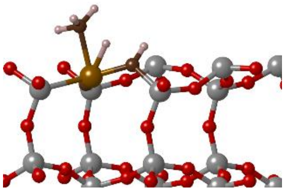	-0.571	3111, 3055, 2974, 2870, 1747, 1460, 1438, 1217, 1128, 947, 804, 651, 529, 389, 208, 158, 115
1.2-1.3b	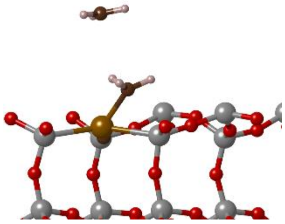	0.245	3102, 3057, 2993, 2801, 1750, 1426, 1393, 1188, 692, 599, 371, 223, 133, 103
1.2-2.6	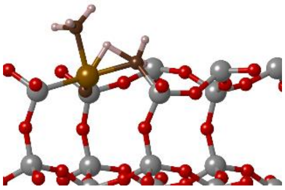	-1.715	3070, 2995, 2923, 1966, 1444, 1429, 1207, 974, 831, 538, 382, 181, 169, 83, 2886, 2707, 1287, 847, 807, 398
2.0-2.1	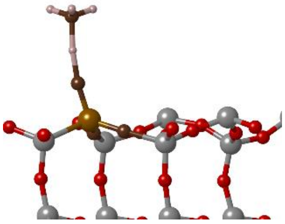	4.194	3073, 3066, 2959, 1428, 1427, 1200, 1187, 1174, 847, 536, 516, 275, 241, 207, 147, 93, 83
2.1-2.2c	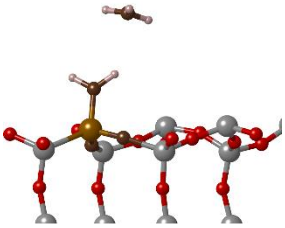	3.227	3218, 3215, 3099, 3047, 2947, 2870, 1391, 1387, 1333, 822, 687, 573, 529, 380, 154, 135, 124, 93, 73, 56

Notation	Structure	Formation energy (eV)	Vibrational frequencies (cm^{-1})
2.1-2.3f	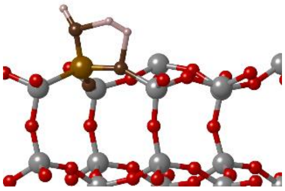	3.540	2855, 1976, 1019, 899, 797, 744, 652, 431, 310, 201, 97
2.2a-2.2b	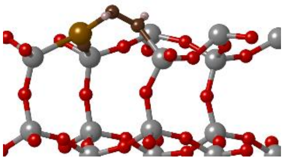	0.198	2993, 2768, 1395, 1259, 993, 881, 645, 549, 408, 307, 246, 160
2.2a-2.3b	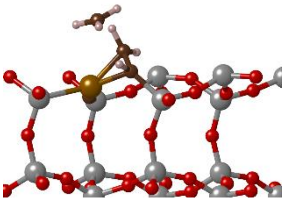	0.478	3227, 3207, 3107, 3043, 2735, 1476, 1393, 1389, 1216, 990, 942, 839, 806, 609, 541, 324, 260, 206, 185, 124, 115, 82, 50
2.2b-2.2c	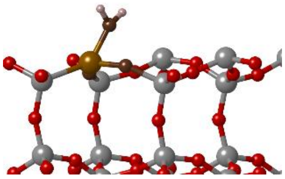	1.422	3134, 3063, 1378, 816, 642, 574, 336, 121
2.2c-2.4b	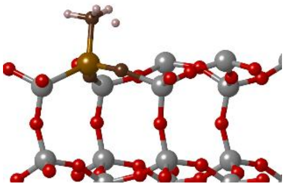	2.054	3261, 3164, 3021, 1417, 1371, 1260, 1095, 629, 620, 531, 449, 194, 138, 31
2.3a-2.4a	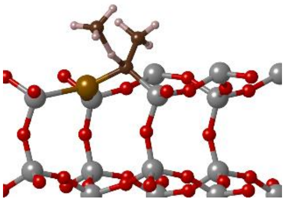	0.298	3171, 3129, 3008, 3001, 2971, 2914, 1474, 1462, 1408, 1405, 1381, 1320, 1126, 1055, 1039, 987, 958, 642, 553, 513, 425, 315, 276, 268, 194, 166, 115, 97, 78
2.3b-2.3c	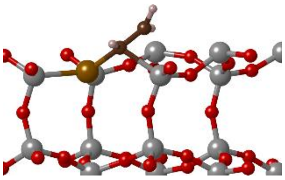	-0.001	3132, 3034, 2794, 1465, 1312, 1196, 937, 919, 857, 616, 406, 341, 285, 151

Notation	Structure	Formation energy (eV)	Vibrational frequencies (cm^{-1})
2.3e-2.3f	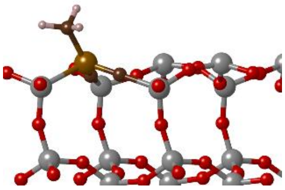	0.226	3041, 3010, 2926, 1431, 1412, 1154, 665, 406, 183, 125, 98
2.3f-2.5a	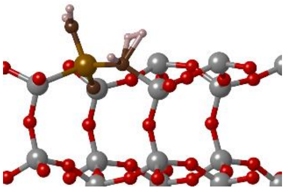	0.561	3027, 2929, 2846, 2432, 1366, 1329, 1124, 972, 842, 701, 656, 639, 524, 455, 308, 138, 86
2.4b-2.5a	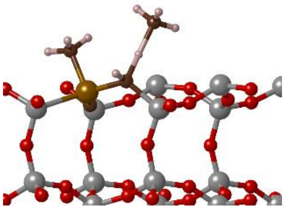	-0.135	3089, 3083, 3080, 3038, 2960, 2921, 2680, 1439, 1420, 1418, 1401, 1269, 1167, 1149, 1100, 923, 744, 724, 689, 592, 573, 540, 492, 460, 404, 259, 236, 158, 143, 122, 111, 105
2.5a-2.5b	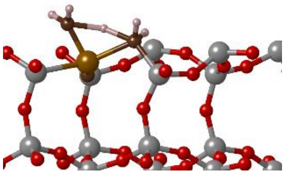	-0.419	3108, 2996, 1791, 1351, 1013, 773, 617, 576, 500, 206, 110, 2886, 2707, 1287, 847, 807, 398
2.5b-2.6	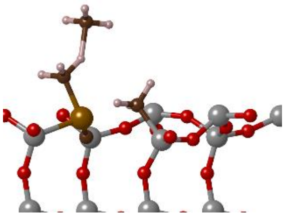	-0.077	3175, 3169, 3045, 3020, 2969, 2918, 2894, 2854, 1829, 1468, 1437, 1407, 1396, 1341, 1341, 1238, 1205, 1027, 877, 857, 809, 718, 706, 565, 551, 520, 449, 367, 340, 334, 305, 260, 215, 185, 171, 89, 71, 57
3.2-3.3a	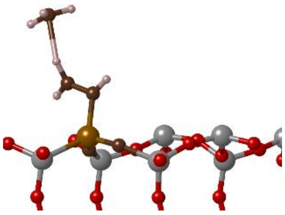	3.709	3218, 3214, 3090, 3069, 3045, 2919, 1513, 1392, 1387, 1361, 1166, 942, 906, 869, 574, 560, 475, 337, 281, 259, 127, 118, 44
3.3a-3.4d	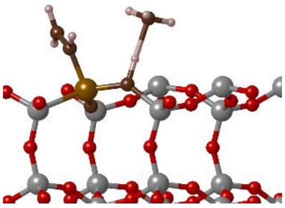	3.238	3202, 3194, 3116, 3030, 3020, 2940, 2319, 1517, 1396, 1389, 1360, 1209, 1006, 952, 933, 911, 732, 661, 580, 423, 399, 350, 309, 246, 178, 157, 105, 70, 46

Notation	Structure	Formation energy (eV)	Vibrational frequencies (cm^{-1})
3.4a-3.5f	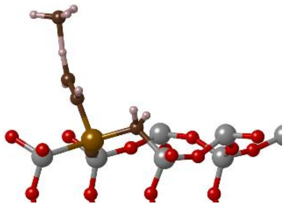	1.929	3166, 3160, 3107, 3014, 2923, 2879, 2817, 1574, 1414, 1405, 1397, 1315, 1182, 1116, 972, 953, 896, 866, 806, 626, 577, 564, 386, 372, 364, 241, 205, 127, 76, 63, 38, 3
3.5a-3.6c	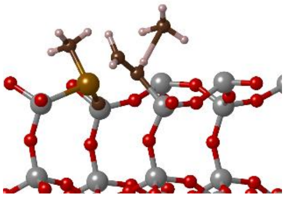	1.759	3218, 3174, 3120, 3069, 3036, 3028, 3014, 2979, 2945, 1527, 1425, 1413, 1390, 1385, 1378, 1371, 1267, 1131, 1027, 959, 921, 672, 626, 594, 566, 546, 429, 370, 359, 308, 286, 212, 185, 151, 149, 133, 80, 67
3.5b-3.5c	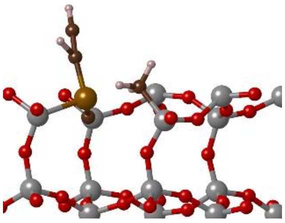	1.816	3343, 2983, 2887, 2798, 2439, 1710, 1476, 1423, 1219, 829, 710, 638, 581, 383, 354, 330, 280, 203, 195, 163, 78, 57, 6
3.5b-3.5f	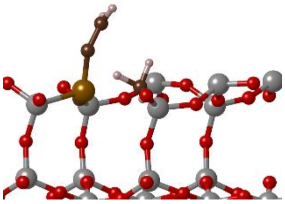	1.468	3067, 3060, 2983, 2956, 2460, 1559, 1508, 1351, 1327, 1172, 899, 863, 751, 691, 610, 523, 430, 373, 335, 237, 168, 84, 48
3.5b-3.5g	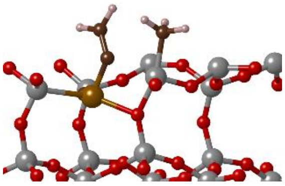	1.692	3118, 3045, 3012, 3004, 2935, 1516, 1465, 1442, 1360, 1246, 950, 828, 818, 791, 562, 477, 433, 379, 336, 302, 265, 206, 70
3.5c-3.6d	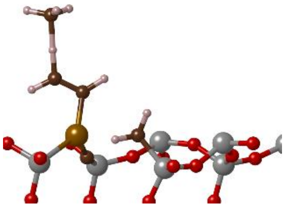	1.774	3154, 3153, 3069, 3024, 3017, 3011, 2987, 2857, 1507, 1453, 1406, 1404, 1356, 1256, 1193, 1092, 1080, 958, 912, 835, 777, 617, 588, 584, 561, 446, 399, 213, 194, 159, 158, 130, 94, 76, 36
3.5h-3.7c	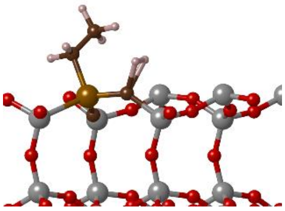	1.743	3133, 3022, 2993, 2964, 2937, 2880, 1471, 1467, 1407, 1383, 1211, 1125, 987, 911, 875, 765, 579, 568, 416, 284, 270, 222, 205, 84, 70, 17

Notation	Structure	Formation energy (eV)	Vibrational frequencies (cm^{-1})
3.6a-3.7b	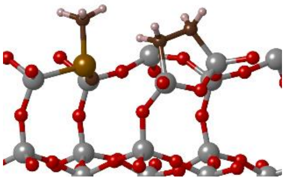	-0.617	3217, 3214, 3065, 3054, 3047, 3019, 2986, 2957, 2940, 2906, 1442, 1428, 1417, 1392, 1390, 1388, 1224, 1217, 1132, 1127, 1044, 887, 739, 666, 630, 612, 610, 541, 518, 437, 314, 185, 162, 104, 94, 81, 77, 64
3.6b-3.7a	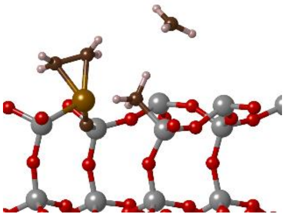	0.283	3208, 3200, 3085, 3034, 3032, 3015, 3001, 2886, 2742, 1495, 1461, 1438, 1399, 1392, 1387, 1213, 1203, 1190, 931, 909, 899, 859, 793, 779, 626, 596, 538, 425, 343, 251, 238, 199, 158, 152, 142, 117, 107, 102, 71, 53, 40
3.7c-3.8a	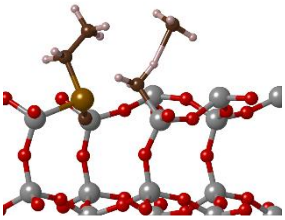	0.492	3200, 3174, 3012, 3006, 2989, 2948, 2947, 2897, 2874, 2836, 2498, 1480, 1479, 1441, 1415, 1402, 1391, 1389, 1380, 1252, 1230, 1225, 1145, 950, 938, 895, 791, 732, 617, 563, 554, 463, 402, 385, 304, 269, 233, 206, 184, 153, 119, 103, 84, 49
