# Supplementary material for: Mechanistic and microkinetic study of non-oxidative methane coupling on a single-atom iron catalyst
Source: Commun Chem. 2020 May 8;3:58. doi: 10.1038/s42004-020-0306-1 (PMC9814405; doi:10.1038/s42004-020-0306-1)
Supplement: Supplementary file 4 — Supplementary Data 2 [file 42004_2020_306_MOESM4_ESM.pdf]

Supplementary data 2. Elementary reactions involved in the microkinetic modelling. * and _g denote surface and gas-phase species, respectively.

#	Reaction	Expression
**1**	1.0-1.1	*_s + CH4_g <-> H-CH3* -> H* + CH3_g
**2**	1.0-1.2	*_s + H2_g <-> H-H* -> H2*
**3**	1.0-2.3a	*_s + CH3_g -> CH3*
**4**	1.0-2.4a	*_s + CH4_g <-> C-H4* -> HCH3*
**5**	1.0-2.4b	*_s + CH4_g <-> CH-H3* -> CH3H*
**6**	1.0-3.2	*_s + C2H2_g -> CHCH*
**7**	1.0-3.4c	*_s + C2H4_g -> H2CCH2*
**8**	1.1-1.2	H* + CH4_g <-> H2-CH3* -> H2* + CH3_g
**9**	1.1-3.3b	H* + C2H2_g -> CHCHH*
**10**	1.1-2.4a	H* + CH3_g -> HCH3*
**11**	1.1-2.4b	H* + CH3_g -> CH3H*
**12**	1.1-2.5a	H* + CH4_g <-> H-CH4* -> CH3H2*
**13**	1.1-3.5i	H* + C2H4_g -> H2CCH2H*
**14**	1.2-1.3a	H2* + CH4_g <-> H2H-CH3* -> H2H* + CH3_g
**15**	1.2-1.3b	H2* + CH4_g <-> HH2-CH3* -> HH2* + CH3_g
**16**	1.2-2.5a	H2* + CH3_g -> CH3H2*
**17**	1.2-2.5b	H2* + CH3_g -> CH2H3*
**18**	1.2-2.6	H2* + CH4_g <-> H2-CH4* -> CH3H3*
**19**	1.2-3.4a	H2* + C2H2_g -> C2H2H2*
**20**	1.2-3.4b	H2* + C2H2_g -> H2C2H2*
**21**	1.2-3.6a	H2* + C2H4_g -> H2C2H4*
**22**	1.2-3.6b	H2* + C2H4_g -> C2H4H2*
**23**	1.3a-3.5c	H2H* + C2H2_g -> C2H2H3*
**24**	1.3a-3.7a	H2H* + C2H4_g -> C2H4H3*
**25**	1.3b-3.5d	HH2* + C2H2_g -> CH3HCH*
**26**	1.3b-3.7b	HH2* + C2H4_g -> CH3H2CH2*
**27**	2.0-2.1	C* + CH4_g <-> CH-CH3* -> CH* + CH3_g
**28**	2.0-2.2c	C* + H2_g <-> H-CH* -> H2C*
**29**	2.0-2.2d	C* + H2_g <-> CH-H* -> CHH*
**30**	2.1-2.2c	CH* + CH4_g <-> CH2-CH3* -> H2C* + CH3_g
**31**	2.1-2.2d	CH* + CH4_g <-> CH3-CH2* -> CHH* + CH3_g
**32**	2.1-2.3e	CH* + H2_g <-> CH-H2* -> CHH2*
**33**	2.1-2.3f	CH* + H2_g <-> CH-H-H* -> CHHH*
**34**	2.2a-2.2b	CH2* <-> C-H-H* -> HCH*
**35**	2.2a-2.3a	CH2* + CH4_g <-> CH3-CH3* -> CH3* + CH3_g
**36**	2.2a-2.3b	CH2* + CH4_g <-> CH3-HCH2* -> HCH2* + CH3_g
**37**	2.2a-3.5a	CH2* + CH3_g -> CH3CH2*
**38**	2.2a-3.5g	CH2* + CH3_g -> CHHCH3*
**39**	2.2b-2.2c	HCH* <-> H-C-H* -> H2C*

**40**	2.2b-2.3c	HCH* + CH4_g <-> CH2H-CH3* -> CH2H* + CH3_g
**41**	2.2b-2.3d	HCH* + CH4_g <-> CH3-H2CH* -> H2CH* + CH3_g
**42**	2.2b-3.5c	HCH* + CH3_g -> C2H2H3*
**43**	2.2b-3.5d	HCH* + CH3_g -> CH3HCH*
**44**	2.2c-2.2d	H2C* <-> HC-H* -> CHH*
**45**	2.2c-2.3e	H2C* + CH4_g <-> CH2-HCH3* -> CHH2* + CH3_g
**46**	2.2c-2.3f	H2C* + CH4_g <-> HCH-HCH3* -> CHHH* + CH3_g
**47**	2.2c-3.5h	H2C* + CH3_g -> H2CCH3*
**48**	2.2c-2.4b	H2C* + H2_g <-> CH2-H2* -> CH3H*
**49**	2.2d-2.3f	CHH* + CH4_g <-> HCH-H2CH2* -> CHHH* + CH3_g
**50**	2.3a-2.3b	CH3* <-> CH2-H* -> HCH2*
**51**	2.3a-2.4a	CH3* + CH4_g <-> CH3-HCH3* -> HCH3* + CH3_g
**52**	2.3b-2.3c	HCH2* <-> H-CH2* -> CH2H*
**53**	2.3b-2.4a	HCH2* + CH4_g <-> HCH3-CH3* -> HCH3* + CH3_g
**54**	2.3c-2.3d	CH2H* <-> HC-H-H* -> H2CH*
**55**	2.3c-3.6d	CH2H* + CH3_g -> C2H3H3*
**56**	2.3d-3.6c	H2CH* + CH3_g -> CH3HCH2*
**57**	2.3e-2.3f	CHH2* <-> HC-H2* -> CHHH*
**58**	2.3e-2.4b	CHH2* + CH4_g <-> HCH2-CH4* -> CH3H* + CH3_g
**59**	2.3f-2.4b	CHHH* + CH4_g <-> CH3-CH4* -> CH3H* + CH3_g
**60**	2.3f-3.6e	CHHH* + CH3_g -> H2CCH3H*
**61**	2.3f-2.5a	CHHH* + H2_g <-> CH3-H2* -> CH3H2*
**62**	2.4b-2.5a	CH3H* + CH4_g <-> H2CH3-CH3* -> CH3H2* + CH3_g
**63**	2.5a-2.5b	CH3H2* <-> CH2-H-H2* -> CH2H3*
**64**	2.5a-2.6	CH3H2* + CH4_g <-> CH3H3-CH3* -> CH3H3* + CH3_g
**65**	2.5a-3.8b	CH3H2* + CH3_g -> CH3H2CH3*
**66**	2.5b-2.6	CH2H3* + CH4_g <-> CH3-CH3H3* -> CH3H3* + CH3_g
**67**	2.5b-3.8a	CH2H3* + CH3_g -> C2H5H3*
**68**	3.2-3.3a	CHCH* + CH4_g <-> C2H3-CH3* -> CHCH2* + CH3_g
**69**	3.2-3.3b	CHCH* + CH4_g <-> HC2H2-CH3* -> CHCHH* + CH3_g
**70**	3.2-3.4a	CHCH* + H2_g <-> C2H2-H2* -> C2H2H2*
**71**	3.3a-3.4c	CHCH2* + CH4_g <-> HC2H3-CH3* -> H2CCH2* + CH3_g
**72**	3.3a-3.4d	CHCH2* + CH4_g <-> H2C2H2-CH3* -> HCCH2H* + CH3_g
**73**	3.3a-3.5f	CHCH2* + H2_g <-> C2H3-H2* -> C2H3H2*
**74**	3.3b-3.4a	CHCHH* + CH4_g <-> H3C2H-CH3* -> C2H2H2* + CH3_g
**75**	3.3b-3.4d	CHCHH* + CH4_g <-> C2H4-CH3* -> HCCH2H* + CH3_g
**76**	3.4a-3.5c	C2H2H2* + CH4_g <-> C2H2H3-CH3* -> C2H2H3* + CH3_g
**77**	3.4a-3.5f	C2H2H2* + CH4_g <-> C2H3H2-CH3* -> C2H3H2* + CH3_g
**78**	3.4b-3.5d	H2C2H2* + CH4_g <-> C2H5-CH3* -> CH3HCH* + CH3_g
**79**	3.4b-3.5e	H2C2H2* + CH4_g <-> HC2H4-CH3* -> H2C2H3* + CH3_g
**80**	3.4c-3.5h	H2CCH2* + CH4_g <-> H2C2H3-CH3* -> H2CCH3* + CH3_g
**81**	3.4c-3.5i	H2CCH2* + CH4_g <-> H3C2H2-CH3* -> H2CCH2H* + CH3_g
**82**	3.4d-3.5i	HCCH2H* + CH4_g <-> H4C2H-CH3* -> H2CCH2H* + CH3_g
**83**	3.4d-3.5h	HCCH2H* + CH4_g <-> H5C2-CH3* -> H2CCH3* + CH3_g

**84**	3.5a-3.6c	CH3CH2* + CH4_g <-> CH3-CH3HCH2* -> CH3HCH2* + CH3_g
**85**	3.5a-3.5d	CH3CH2* <-> CH3-H-CH* -> CH3HCH*
**86**	3.5b-3.5c	CH2CH3* <-> CH-H-CH3* -> C2H2H3*
**87**	3.5b-3.5g	CH2CH3* <-> C-H2CH3* -> CHHCH3*
**88**	3.5b-3.5f	CH2CH3* <-> CH2-H-H2C* -> C2H3H2*
**89**	3.5b-3.6d	CH2CH3* + CH4_g <-> CH3-C2H3H3* -> C2H3H3* + CH3_g
**90**	3.5c-3.6d	C2H2H3* + CH4_g <-> C2H3H3-CH3* -> C2H3H3* + CH3_g
**91**	3.5d-3.6c	CH3HCH* + CH4_g <-> CH3HCH2-CH3* -> CH3HCH2* + CH3_g
**92**	3.5e-3.6c	H2C2H3* + CH4_g <-> C2H6-CH3* -> CH3HCH2* +CH3_g
**93**	3.5e-3.6a	H2C2H3* + CH4_g <-> CHCH5-CH3* -> H2C2H4* + CH3_g
**94**	3.5f-3.6d	C2H3H2* + CH4_g <-> CH2CH4-CH3* -> C2H3H3* + CH3_g
**95**	3.5f-3.6b	C2H3H2* + CH4_g <-> CH3CH3-CH3* -> C2H4H2* + CH3_g
**96**	3.5h-3.6e	H2CCH3* + CH4_g <-> CH4CH2-CH3* -> H2CCH3H* + CH3_g
**97**	3.5h-3.7c	H2CCH3* + H2_g <-> C2H5-H2* -> H2CCH3H2*
**98**	3.5i-3.6b	H2CCH2H* + CH4_g <-> CH5CH-CH3* -> C2H4H2* + CH3_g
**99**	3.5i-3.6e	H2CCH2H* + CH4_g <-> CH6C-CH3* -> H2CCH3H* + CH3_g
**100**	3.6a-3.7b	H2C2H4* + CH4_g <-> CH3-CH3H2CH2* -> CH3H2CH2* + CH3_g
**101**	3.6b-3.7c	C2H4H2* + CH4_g <-> C2H7-CH3* -> H2CCH3H2* + CH3_g
**102**	3.6b-3.7a	C2H4H2* + CH4_g <-> C2H4H3-CH3* -> C2H4H3* + CH3_g
**103**	3.6c-3.7b	CH3HCH2* + CH4_g <-> CH3H2CH2-CH3* -> CH3H2CH2* + CH3_g
**104**	3.6d-3.7a	C2H3H3* + CH4_g <-> CH3-C2H4H3* -> C2H4H3* + CH3_g
**105**	3.6e-3.7c	H2CCH3H* + CH4_g <-> CH6CH-CH3* -> H2CCH3H2* + CH3_g
**106**	3.7a-3.8a	C2H4H3* + CH4_g <-> CH3-C2H5H3* -> C2H5H3* + CH3_g
**107**	3.7b-3.8b	CH3H2CH2* + CH4_g <-> CH3H2CH3-CH3* -> CH3H2CH3* + CH3_g
**108**	3.7c-3.8a	H2CCH3H2* + CH4_g <-> C2H8-CH3* -> C2H5H3* + CH3_g
